# A deafness-blindness syndrome results from *ATF6*-based disruption of the unfolded protein response

**DOI:** 10.1172/JCI188708

**Published:** 2025-02-03

**Authors:** Yuvraj Joshi, Jeffrey N. Savas

**Affiliations:** Ken and Ruth Davee Department of Neurology and Knowles Hearing Center, Feinberg School of Medicine, Northwestern University, Chicago, Illinois, USA.

## Abstract

Sensorineural hearing loss (SNHL) is the most prevalent form of permanent hearing impairment, arising from factors such as aging, exposure to loud noise, disease, ototoxic medications, and genetic mutations. Despite extensive research, effective treatments or cures for SNHL remain elusive. In this issue of the *JCI*, Lee et al. reveal a link between mutations in *ATF6* and SNHL in patients with achromatopsia. The study also shows that *Atf6*-deficient (*Atf6^–/–^*) mice exhibit disorganized stereocilia and age-related loss of outer hair cells. Additionally, the researchers show that *Atf6* is critical for cochlear hair cell function. Mice lacking *Atf6* expression experienced ER stress, which ultimately led to SNHL. Collectively, these findings enhance our understanding of the emerging role of protein homeostasis and ER stress in the pathogenesis of SNHL.

## The cochlea is a high-fidelity auditory organ

The cochlea is a complex sensory organ that converts sound into meaningful auditory signals in a frequency- and intensity-dependent manner (1). It consists of three fluid-filled compartments — the scala vestibuli, scala media, and scala tympani — arranged around the bony modiolus. This design enables the cochlea to process sound with exceptional precision. The organ of Corti, is at the core of cochlear function. It is bathed with scala media and harbors two types of sensory cells — inner hair cells (IHCs) and outer hair cells (OHCs). Hair cells and their mechanosensitive stereocilia, along with other supporting structures such as the stria vascularis, tectorial membrane, and Reisner’s membrane, are all critical for hearing. Like all highly complex, high-fidelity instruments, the cochlea is vulnerable to both intrinsic and extrinsic factors, which can lead to temporary or permanent functional impairment. Therefore, cellular systems essential for maintaining cell health, function, and survival are crucial in complex organs like the cochlea to support hearing.

## Mutations in *ATF6* cause deafness-blindness syndrome

In this issue of the *JCI*, Lee and co-authors initially sought to explore whether patients lacking functional activating transcription factor 6 (*ATF6*) had any disorders beyond vision loss (2). ATF6 is a key transcription factor for maintaining ER function and protein homeostasis and is important for inducing the unfolded protein response (UPR) (3). Previous evidence shows that people carrying loss-of-function *ATF6* disease alleles have congenital vision loss, achromatopsia, and cone-rod dystrophy (4, 5). Notably, the authors now report that patients carrying the c.970C > T *ATF6* variant also have hearing loss. Using pure tone audiometry tests, the authors showed that the hearing loss was progressive and binaural, suggesting patients have more difficulty in hearing from both ears at middle age. Thus, seeing that the patients present with both blindness and deafness, individuals with *ATF6* mutations can now be classified under the syndromic hearing loss category. The most studied and frequent cause of heredity deafness-blindness remains Usher syndrome (US), which is caused by mutations in multiple genes, including *MYO7A*, *USH1C*, *CDH23*, *PCDH15*, and *SANS* (type 1); *USH2A*, *ADGRV1*, and *WHRN* (type 2); and *CLRN1* (type 3). Notably, CDH23 and PCDH15 proteins play essential roles in maintaining stereocilia bundle organization (6–10). It was reported that mutations in *CIB2*, which encodes a calcium-binding protein, are also associated with US (11). However, biallelic loss-of-function variants in *CIB2* cause recessive, nonsyndromic hearing loss but not US (12). The implication is that the phenotype resulting from the *ATF6* mutation closely resembles that of US, showing similar clinical or physiological characteristics. However, despite these phenotypic similarities, the underlying molecular or cellular mechanisms driving the *ATF6* mutation phenotype differ fundamentally from those involved in US, suggesting a somewhat distinct pathophysiological process.

## Impaired proteostasis as a critical link in sensorineural hearing loss

Sensorineural hearing loss (SNHL) encompasses any form of hearing loss resulting from damage to the cochlea, auditory nerve, or central auditory system. It is the most prevalent congenital sensory disorder and is classified into genetic and acquired forms. Genetic SNHL is further divided into nonsyndromic (70%) and syndromic (30%) types. Nonsyndromic hearing loss occurs in isolation, without any additional symptoms or disabilities. In contrast, syndromic hearing loss is characterized by additional symptoms that affect other parts of the body. Noise-induced hearing loss (NIHL) is a major cause of SNHL in adults, with an estimated 16% of cases worldwide resulting from occupational exposure (13). The interplay between genetic factors and environmental conditions can contribute to the development of NIHL. A set of genes associated with the predisposition to NIHL susceptibility have been identified and are involved in oxidative stress, stereocilia, protein folding, ion homeostasis, DNA repair, apoptosis, and others. Lee and co-authors provide compelling evidence that mice lacking *Atf6* expression have a substantial induction of the UPR, especially the PRK-like ER kinase (PERK) arm, since several ATF6 target genes are altered, including *Hsp90b1*, which is involved in the ER-associated protein degradation (ERAD) pathway (2).

The regulation of protein homeostasis, or “proteostasis,” refers to the processes by which cells maintain the proper balance of protein synthesis, folding, and degradation. The proteostasis network, comprising the proteins responsible for maintaining proteostasis, is crucial for ensuring cellular function and health (14). Proteotoxicity is the disruption of proteostasis, which occurs as a result of a variety of factors such as translational errors, protein misfolding, or protein damage. The imbalance in protein quality control mechanisms can lead to the accumulation of damaged or misfolded proteins, which can impair cellular function, promote cellular stress and aging, and contribute to many disorders, including neurodegeneration and hearing loss (15–20).

Notably, exposure to loud noise causing hearing loss severely unbalances the cochlear proteome by causing hundreds of proteins to accumulate (21). This process, in turn, activates the proteostasis network by increasing the expression of heat shock protein (HSP) chaperones (22), including the cytosolic HSPs Hsp90aa1 and Hsp90ab1, as well as Hsp90b1, which localizes to the ER (23). Aging is another key factor in the development of SNHL. Age-related hearing loss (ARHL), or presbycusis, is a progressive, common, and irreversible condition that is likely caused by multiple processes and pathways. However, further evidence of impaired proteostasis in ARHL is provided by single-cell transcriptomics, which revealed that *Hsp90aa1* is one of the genes with the most elevated expression in cochlear intermediate cells of the stria vascularis during aging (24).

## *Atf6*-KO mice show cochlear pathology

To further investigate the role of *Atf6* in hearing, Lee and colleagues recorded auditory brainstem responses (ABRs) to assess cochlear function in *Atf6^–/–^* mice (2). At P14, no notable differences in ABR thresholds were observed between *Atf6^–/–^* and *Atf6^+/+^* mice. However, by 2 months of age, ABR thresholds were elevated in both sexes of *Atf6^–/–^* mice. Thus *Atf6^–/–^* mice had slow progressive hearing loss resembling the hearing loss observed in the patients carrying *ATF6* disease alleles, albeit at relatively dissimilar respective ages. Otoacoustic emissions, which reflect the normal function of OHCs, were severely reduced in patients but were not assessed in *Atf6^–/–^* mice.

Deafness caused by gene mutations is often associated with morphological disorganization of stereocilia bundles, which are essential for converting sound into electrical signals that facilitate hearing (25). Lee and authors evaluated sensory hair cell morphology using immunofluorescence in cochlear whole mounts from *Atf6^–/–^* mice (2). The hair cell marker myosin VII and the filamentous actin dye phalloidin were used to visualize the hair cells and stereocilia, respectively. They observed loss and disrupted arrangement of OHCs in the basal region of the cochlea. However, the number of OHCs in the apical region and the number of IHCs across the entire cochlea were normal. Actin labeling revealed disorganized and frayed stereocilia bundles in the hair cells of the cochlea from *Atf6^–/–^* mutants. The results suggest that deformation of stereocilia and OHC death are the cause of hearing loss in *Atf6^–/–^* mice. The authors could have used high-resolution scanning electron microscopy to further validate the deformed stereocilia morphology at nanometer resolution. Since the diameter of stereocilia is less than a wavelength of light, light microscopy is not able to reliably detect subtle changes in its ultrastructural morphology.

## Impaired ER stress response in *Atf6^–/–^* cochlea

To gain further insight into the mechanisms underlying hearing loss in *Atf6^–/–^* mice, Lee and co-authors performed bulk RNA-Seq analysis (2). They focused on selected differentially expressed genes (DEGs) and performed gene ontology (GO) analysis to identify groups of functionally related DEGs. GO analysis revealed that genes associated with ER stress, ion homeostasis, actin filaments, and neuronal death were altered in *Atf6^–/–^* cochlea. ER stress is known to activate the UPR, a protective mechanism to restore homeostasis and ensure proper protein folding by preventing the accumulation of misfolded proteins. The UPR restores homeostasis by upregulating ER protein chaperones such as Hsp90b1, to refold the mishandled proteins, or by degrading misfolded proteins. Notably, the authors did not observe changes in genes related to autophagy, the proteasome, or oxidative stress. Nevertheless, several DEGs encoding stereocilia bundle–associated proteins were observed, which may explain the disorganized stereocilia. Despite enrichment of multiple ion channel ontologies in *Atf6^–/–^* cochlea, no differences in the overall mean expression levels of channel-related gene sets were observed. However, alterations of several DEGs encoding ion channels were identified.

## Conclusion and future directions

**Figure 1:**
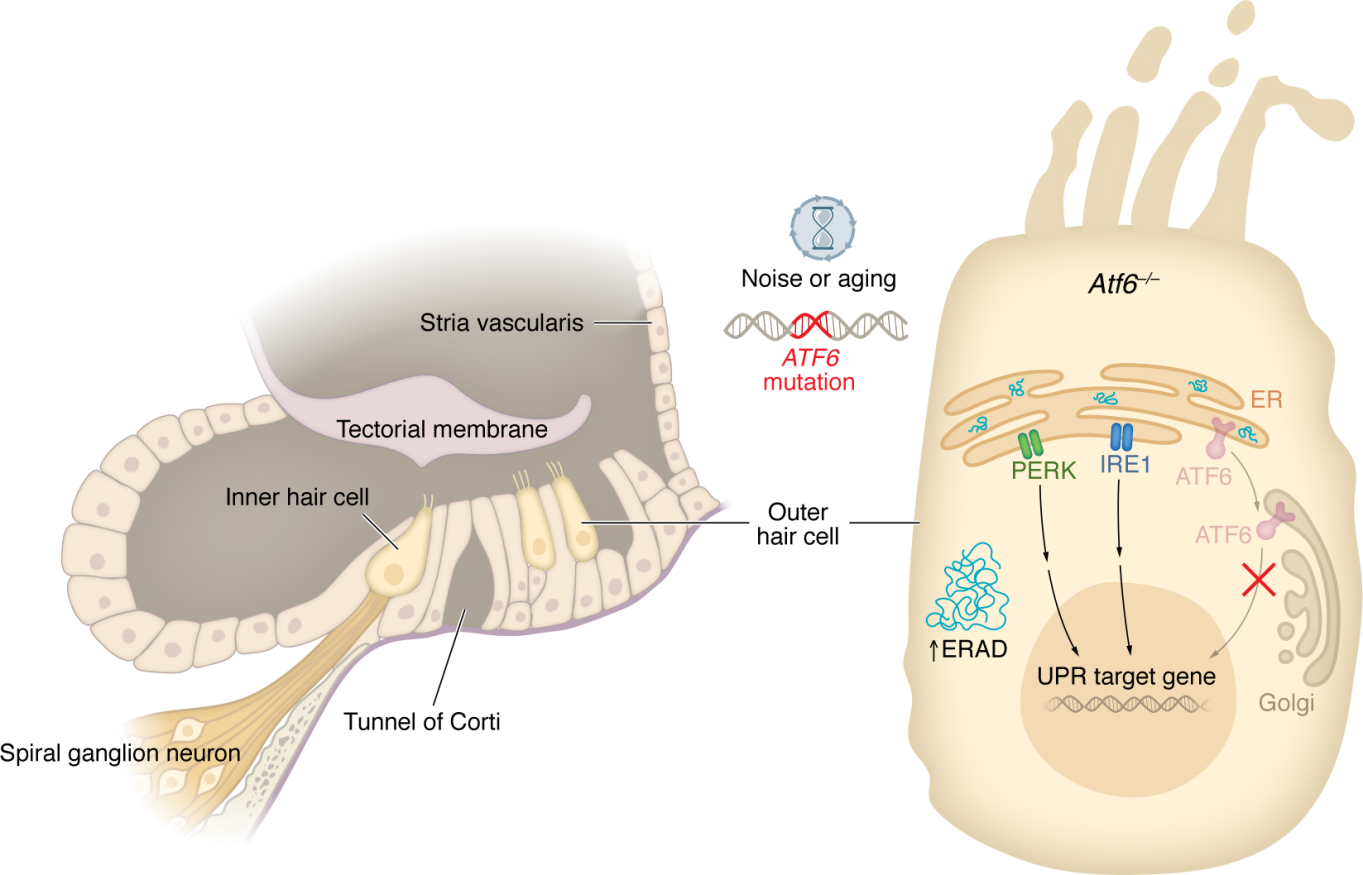
A deafness-blindness syndrome results from ATF6-mediated disruption of the UPR, leading to altered hair cell stereocilia and cell death. In human and mouse cochleae lacking expression of ATF6, OHC dysfunction and death occur, with hearing loss progressively worsening in middle age. In the absence of *Atf6* expression in mice, genes involved in ER stress, the UPR, and actin filaments (e.g., stereocilia) are dysregulated. ATF6 loss results in activation of the UPR and upregulation of genes involved in the ERAD pathway. These findings suggest that the human and mouse phenotypes are linked to compromised cochlear proteostasis. However, the exact mechanism connecting altered gene expression, stereocilia deformation, and OHC death remains unclear. IRE1, inositol-requiring enzyme 1.

The findings by Lee et al. (2) underscore the therapeutic potential for targeted strategies aimed at enhancing the proteostasis network to treat or prevent hearing loss. One promising approach for managing SNHL could be to treat patients with medications that upregulate the UPR to reduce ER stress. Despite the unresolved questions, the study offers insights of value. These findings not only deepen our understanding of a newly identified human disorder but also provide important genetic evidence supporting the role of proteostasis in hearing loss (Figure 1).

## Version 1 02/03/2025

Electronic publication
